# The Association between Energy Balance-Related Behavior and Burn-Out in Adults: A Systematic Review

**DOI:** 10.3390/nu12020397

**Published:** 2020-02-02

**Authors:** Yanni Verhavert, Kristine De Martelaer, Elke Van Hoof, Eline Van Der Linden, Evert Zinzen, Tom Deliens

**Affiliations:** 1 Department of Movement and Sport Sciences, Vrije Universiteit Brussel, Pleinlaan 2, 1050 Brussels, Belgium; kdmartel@vub.be (K.D.M.); eline.van.der.linden@vub.be (E.V.D.L.); tom.deliens@vub.be (T.D.); 2 Faculty of Social and Behavioral Sciences, Utrecht University, Heidelberglaan 1, 3584 CS Utrecht, The Netherlands; 3 Department of Psychology, Vrije Universiteit Brussel, Pleinlaan 2, 1050 Brussels, Belgium; elke.van.hoof@vub.be

**Keywords:** mental health, emotional exhaustion, cynicism, professional efficacy, physical activity, sedentary behavior, dietary behavior

## Abstract

Although it is believed that physical activity, sedentary, and dietary behavior (i.e., energy balance-related behavior) may decrease the risk of burn-out, the association between both is currently not well understood. Therefore, the aim of this systematic review was to synthesize studies investigating the relationship between energy balance-related behavior and burn-out risk. A systematic literature search was conducted in four databases, resulting in 25 included studies (ten experimental and 15 observational studies). Nine out of ten experimental studies showed that exercise programs were effective in reducing burn-out risk. Fourteen out of fifteen observational studies found a negative association between physical activity and burn-out risk, whereas one study did not find a relation. Two of the 15 observational studies also showed that being more sedentary was associated with a higher burn-out risk, and two other studies found that a healthier diet was related to a lower burn-out risk. No experimental studies were found for the latter two behaviors. It can be concluded that physical activity may be effective in reducing burn-out risk. The few observational studies linking sedentary and dietary behavior with burn-out risk suggest that being more sedentary and eating less healthy are each associated with higher burn-out risk. More high-quality research is needed to unravel the causal relationship between these two behaviors and burn-out risk.

## 1. Introduction

Over recent decades, the prevalence—but also the recognition—of burn-out has increased enormously [1,2]. Burn-out often leads to absenteeism and presenteeism at work [3,4,5], and so it is an increasing concern in today’s workplaces [6]. In the European Union, work-related stress costs EUR 25.4 billion annually, whereas globally, burn-out and stress cost more than USD 300 billion every year [7,8].

Burn-out can be defined as a “prolonged response to chronic emotional and interpersonal stressors on the job, determined by three dimensions: emotional exhaustion, cynicism or depersonalization, and professional (in)efficacy or personal accomplishment” [9]. People experiencing burn-out are mainly mentioning feelings of mental and physical exhaustion, low mood and lack of energy, and therefore emotional exhaustion is seen as the key component of burn-out [1,9,10]. To have better insight into the relationships people have with their job, it is also important to include the other two dimensions. Cynicism refers to the cognitive distance burned out people are taking from their job, and professional (in)efficacy refers to the feeling of being incompetent at work [9,11]. 

To reduce the high—and increasing—incidence and prevalence of burn-out, effective interventions are needed. In a systematic review by Awa et al. [12], it was concluded that a lot of interventions, such as relaxation training and task restructuring at work, are effective, but after several months the positive effects are diminished. This shows the need to develop interventions that are effective in the long term. It has been argued that energy balance-related behavior—including physical activity, sedentary and dietary behavior—may play an important role in preventing and/or curing burn-out. Although physical (in)activity and diet have been associated with mental health, depression and anxiety [13,14,15], their link with burn-out is unclear. There are several reasons why energy balance-related behavior may be effective to reduce and prevent burn-out. 

Physical activity is defined as “any bodily movement produced by skeletal muscles and which requires energy expenditure” [16]. The benefits of physical activity and exercise are enormous. Besides the well-known cardiovascular adaptations, they can increase cerebral blood flow, upregulate neurotrophic factors (e.g., brain-derived neurotrophic factor (BDNF)), support cognitive function and improve executive functions (e.g., planning and sequencing) [17,18,19]. Furthermore, physical activity can facilitate taking psychological distance from work, which reduces job stress and increases job performance [20,21]. As people with burn-out have decreased BDNF-levels, increased job stress, and decreased cognitive function, the abovementioned benefits of physical activity may reduce or even prevent burn-out [22,23,24]. In a systematic review by Naczenski et al. [1], which included ten studies, it was concluded that physical activity may be effective in reducing burn-out levels, showing a possible causal relationship between both. On the other hand, a systematic review and meta-analysis by Ochentel et al. [6] did not find clear (statistical) evidence that exercise therapy is effective in reducing burn-out levels. It should be said, though, that the majority of the included studies in the meta-analysis reported significant differences between the intervention and control groups. Moreover, only four studies were meta-analyzed, making it difficult to make reliable statistical statements.

Sedentary behavior is defined as “any waking behavior characterized by an energy expenditure ≤ 1.5 METs (Metabolic Equivalent of Task) while in a sitting, reclining or lying posture” [25]. Sedentary behavior is associated with physical (in)activity [26], but there is still a clear distinction between both [27,28]. A systematic review by Rezende et al. [29] concluded that sedentary behavior may be a determinant of health, independently of physical activity. In addition, van der Ploeg et al. [27] indicated that sedentary behavior and physical (in)activity should be targeted at the same time in public health strategies, while the two earlier mentioned systematic reviews [1,6], only included studies assessing the link between physical activity and burn-out, without taking sedentary behavior into account. It has been suggested that sedentary behavior may influence mental performance and mental health. Watching television, for example, is associated with decreased executive functioning and decreased cognitive performance [30,31]. Furthermore, Engeroff et al. [32] found that BDNF-levels are negatively associated with sedentary behavior. In a systematic review by Teychenne et al. [33] it was suggested that sedentary behavior is associated with a higher risk of depression, while an experimental study demonstrated that increased sedentary time may result in decreased mood [34]. 

Dietary behavior is another component of energy balance-related behavior and includes aspects such as dietary intake, diet quality and dietary patterns. Diet may also play a role in reducing and preventing burn-out, as it exerts a certain influence on neurotransmitters and neurotransmission. Research showed that the function and levels of neurotransmitters are different in people with burn-out [35,36]. Tops et al. [35] found that people experiencing burn-out are showing a low serotonergic and a low dopaminergic function. Furthermore, low exhaustion is associated with higher neurotransmitter levels, such as norepinephrine, dopamine and acetylcholine, compared to people with moderate exhaustion [36]. It should be mentioned that, in the latter study, a comparison with people experiencing high exhaustion could not be made due to the lack of highly exhausted people. Previous research has already demonstrated the mediating role of neurotransmission in the relationship between diet and mental health. For example, the administration of tryptophan increases brain serotonin synthesis, which, in turn, influences serotonin-dependent brain functions such as mood [37]. Tryptophan can be found in foods such as poultry, milk and some seeds. Likewise, the administration of tyrosine increases the production and release of dopamine and norepinephrine [37], which was found to be diminished in exhausted people. Tryptophan can be found in foods such as poultry, milk and some seeds. Likewise, the administration of tyrosine increases the production and release of dopamine and norepinephrine [37], which was found to be diminished in exhausted people. An increased production and release of this amino acid can be useful in enhancing performance during highly stressful situations. Tyrosine can be found in foods such as dairy, meat and fish. Secondly, studies showed that glucose administration and dietary carbohydrates enhance cognitive performance [38,39,40], while Chung et al. [41] suggested that also a mixed-grain diet can be beneficial for cognitive performance. This latter study also found beneficial effects of mixed-grain diet on plasma BDNF levels, which are decreased in people with burn-out. Lastly, omega-3-supplementation is associated with mood state, which results in an increase in feelings of vigor and a decrease in feelings of anger, anxiety, fatigue, depression and confusion [42]. The abovementioned physiological mechanisms hypothesize the preventative and healing functions of energy balance-related behavior towards burn-out. 

It is clear that—given the rise in incidence and prevalence of burn-out—effective interventions are urgently needed. Improving energy balance-related behavior may be a promising strategy to counter burn-out. Although two recent (contradicting) systematic reviews [1,6] on the single association between physical activity and burn-out have been published, to date, no overview of studies investigating the relationship between energy balance-related behavior from a holistic point of view (including physical activity, sedentary and dietary behavior) and burn-out is available. Therefore, the aim of the present systematic review is to synthesize studies investigating the association between energy balance-related behavior and burn-out.

## 2. Materials and Methods 

This review is registered in PROSPERO with registration number: CRD42019124458.

### 2.1. PICO Statement

The present systematic review investigates the association between energy balance-related behavior (i.e., physical activity, sedentary and dietary behavior) (=exposure or intervention) and burn-out (=outcome) in adults (=population).

### 2.2. Databases and Key Words

Following the PRISMA guidelines for conducting systematic reviews [43], a search was conducted in PubMed, Web of Science, PsycINFO and Embase using the following search terms: “energy balance-related behavior”, “energy balance”, “energy expenditure”, “physical (in)activity”, “physically (in)active”, “exercise”, “training”, “sport”, “moving”, “work-out”, “leisure (time) activity”, “walking”, “biking”, “(in)active lifestyle”, “lifestyle (related) activity”, “household activity”, “housework”, “gardening”, “active transport”, “transportation”, “sedentary”, “sitting”, “lying down”, “diet”, “food”, “eating”, “nutrition”, “caloric intake”, “energy intake”, “burn-out”, “affective disorder”, “adaptive disorder”, “common mental disorder”, “psychological discomfort”, “psychological stress”, “psychological health”, “psychological illness”, “psychological fatigue”, “job stress”, “toxic stress”, “chronic stress”, “work stress”, “occupational stress”, “occupational health”, “exhaustion”, “mental fatigue”, “mental illness”; “mental disorder”, “well-being”, “emotional burden”, “depersonalisation”, “personal accomplishment”, “cynicism”, “inefficacy”. The PICO/PECO method [44] was used to structure and combine key words using Boolean terms. Wildcards were used for plural, other spelling and to cover British and American English equivalents. 

### 2.3. Eligibility Criteria

Results were limited to original English-language articles published in full-text format in peer reviewed journals until January 2019, assessing the direct relationship between energy balance-related behavior (i.e., physical activity, sedentary and dietary behavior) and burn-out or one or more of its components (i.e., emotional exhaustion, cynicism and inefficacy) in working adults between 18 and 65 years old. Meta-analyses, systematic reviews, methodological papers, congress proceedings, meeting abstracts and case studies were excluded from the search. Studies were also excluded when investigating other affective disorders than burn-out (such as depression, bipolar disorders and anxiety disorders), investigating the influence of an intervention including more than only physical activity (such a mediation exercises, workshops, etc.) on burn-out levels, including non-working adults such as people in prison, students, athletes, etc., investigating the association between burn-out and disordered eating behaviors (such as emotional eating, uncontrolled eating, etc.), or investigating the association between burn-out and alcohol.

### 2.4. Selection of Studies

After completing the search in each database, all references were imported into EndNote (=bibliographic software program) and then exported to Rayyan (=bibliographic software program designed to facilitate systematic reference selection), in which the study selection was conducted. The study selection included the screening of titles, abstract and full-texts and conducting a forward and backward search. The search and study selection were conducted in January 2019 by two researchers independently from each other (YV and EVDL). Any doubts or disagreements between the two researchers were discussed with a third researcher (TD). The followed methodology was reviewed and approved by the head of the university library (KA). When important information in the articles was missing, authors were contacted via e-mail.

### 2.5. Quality Assessment

The ‘Standard quality assessment criteria for evaluating primary research papers from a variety of fields’ [45] was used to assess the methodological quality of the included studies. The checklist consists of 14 items which were given a certain score depending on whether or not the specific criterion was met (“no” = 0, “partial” = 1, “yes” = 2). Depending on the study design, some items were not applicable and were therefore scored as ‘not applicable (N/A)’ and excluded from the calculation of the total score. The total sum was calculated by summing the total number of “yes” multiplied by 2 and the total number of “partials” multiplied by 1. The total possible sum was calculated as follows: 28 − (number of ‘N/A’ × 2). Lastly, the summary score was calculated by dividing the total sum by the total possible sum.

## 3. Results

**Figure 1:**
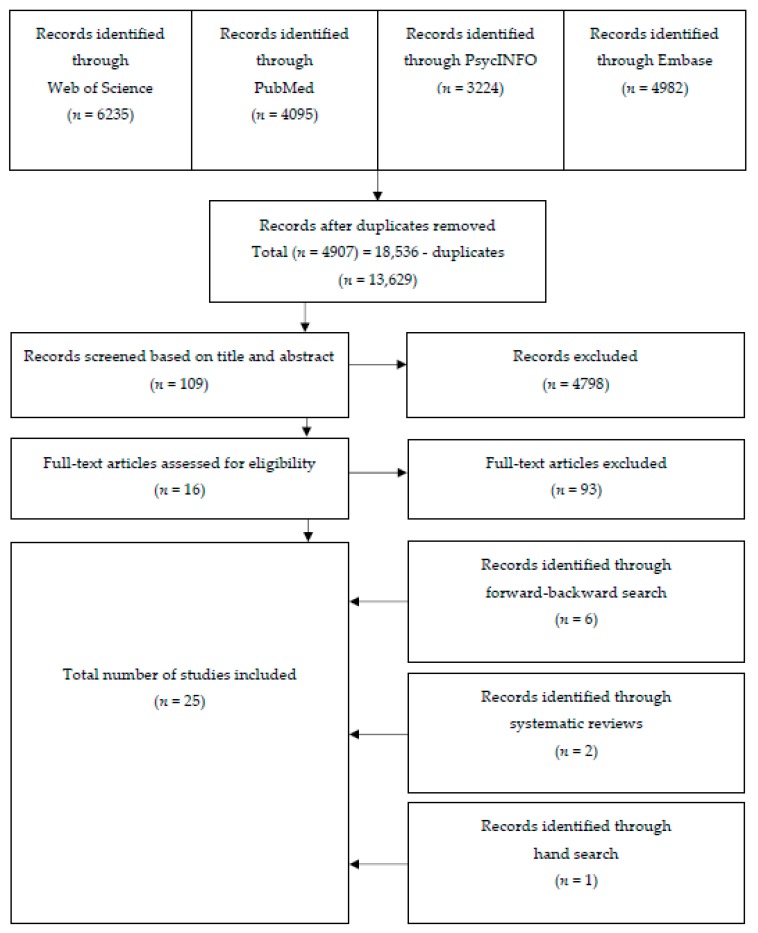
Flow chart of the systematic search.

In total, 18,536 articles were found. When removing all duplicates, 4907 articles remained. Titles and abstracts were screened for eligibility. Of the 109 remaining articles, sixteen studies met the inclusion criteria. In addition, a forward and backward search was performed through which we identified six more studies. Another two articles were included after screening the reference lists of two previously published systematic reviews investigating the association between physical activity and burn-out [1,6]. One more article was obtained through hand search [46]. So, in total, 25 articles were included in the final synthesis. The flow chart of the search process is displayed in Figure 1.

The 25 included studies consisted of ten experimental and 15 observational studies. The experimental studies included five randomized controlled trials (RCTs), one randomized clinical trial, one quasi experimental and three pre-experimental studies. An overview of the included experimental studies is presented in Table 1. The observational studies included six longitudinal and nine cross-sectional studies. An overview of the included observational studies is presented in Table 2.

### 3.1. Physical Activity

All 25 studies assessed the relationship between physical activity and burn-out. 

Nine out of ten experimental studies found a positive effect of physical activity on risk of burn-out. More specifically, five studies found a reduction in risk of burn-out in general (i.e., all three dimensions combined) due to physical activity (reduction ranging from 6.9% to 41.3%) [49,52,53,54,55]. Two studies assessed the effect of physical activity on all three dimensions of burn-out separately, reporting a positive effect on emotional exhaustion (−33.4% and −10%) and cynicism (−33.3% and −4%), but not on personal accomplishment [51,56]. However, one other study also assessed the effect of physical activity on emotional exhaustion, cynicism and personal accomplishment separately and did not find a significant effect on any dimension [50]. Two of the ten experimental studies assessed the effect of physical activity on emotional exhaustion only and showed a significant improvement in emotional exhaustion (−21.6% and −10%) [47,48]. 

Four experimental studies also conducted follow-up measurements, with three studies reporting a decrease in risk of burn-out three and six months after the physical activity intervention [47,53,56]. The fourth study equally showed a decrease after six months and this maintained after 12 months [52]. Four studies compared physical activity interventions with other interventions such as multimodal rehabilitation and basic care, but no significant differences in the decrease in burn-out risk between these interventions were reported [49,52,54,56]. 

All 15 observational studies assessed the relationship between physical activity and risk of burn-out, or one of its components. Fourteen observational studies found a negative association between physical activity and risk of burn-out, or one of its components, and one did not find a relationship. More specifically, ten studies assessed the relationship between physical activity and risk of burn-out generally [21,61,63,64,66,67,68,69], of which nine found a negative association and one did not find a significant relation between both [65]. One observational study assessed both the relationship between physical activity and risk of burn-out and all three dimensions separately and found negative associations between physical activity and risk of burn-out, emotional exhaustion and cynicism and a positive association between physical activity and professional efficacy [57]. Four other studies found a negative association between physical activity and emotional exhaustion [46,58,60,70] and one of them also found a positive association between physical activity and cynicism [46]. Lastly, one of 15 observational studies concluded that the association between physical activity and risk of burn-out was stronger in workers with a sedentary job [58].

### 3.2. Sedentary Behavior

Two of the 15 observational studies including physical activity also assessed the relationship between sedentary behavior and risk of burn-out. Both studies found a positive association with risk of burn-out [67,68]. 

### 3.3. Dietary Behavior

Besides physical activity, two of the observational studies also examined the link between diet and risk of burn-out. In both studies diet was measured by a single question. The study by Alexandrova-Karamanova et al. [46] measured fast food consumption by asking how many times in a week people ate fast food, and concluded that fast food consumption was positively associated with risk of burn-out. In the study by Gorter et al. [69] dietary behavior was measured by asking how many healthy diets participants consumed during workdays, and found that the amount of healthy diets during workdays and the risk of burn-out were negatively associated.

### 3.4. Quality Assessment of the Included Studies

According to the “Standard quality assessment criteria for evaluating primary research papers from a variety of fields” [45], the mean article quality score was 0.82 ± 0.10 out of a total of 1 (Table 3). Eleven articles scored below the mean score with a minimum score of 0.61. Fourteen articles scored above the mean score with a maximum score of 0.95.

## 4. Discussion

In this systematic review, an overview of studies investigating the association between energy balance-related behavior and burn-out is provided. In total, 25 studies were found, of which 21 assessed the relationship between physical activity and risk of burn-out, two studies investigated the association between physical activity, sedentary behavior and the risk of burn-out, and two assessed the link between physical activity, dietary behavior and risk of burn-out. No articles using a holistic approach—i.e., investigating the relationship between energy balance and energy balance-related behavior as a whole and risk of burn-out—were found. Nevertheless, it is important to use a combined approach to gain more insight regarding, e.g., which behavior may have a bigger impact on burn-out risk. Moreover, as previously mentioned, van der Ploeg et al. [27] indicated that sedentary behavior and physical (in)activity should be targeted at the same time in public health strategies. The present systematic review, for example, shows that the association between physical activity and risk of burn-out was stronger in workers with a sedentary job, showing a possible interaction between both physical activity and sedentary behavior in relation to burn-out risk [58]. A similar interaction between both behaviors, but with mortality as the outcome measure, was found in a large-scale meta-analysis (including over one million men and women), where high levels of moderate intensity physical activity (about 60–75 min per day) eliminated the increased risk of death associated with high sitting time [71]. In addition, previous studies showed that increased screen time was associated with an overall poor diet quality [72,73]. The above indicates possible triangular interactions, again highlighting the importance of combining all energy balance-related behaviors when investigating their association with burn-out risk.

Furthermore, despite the fact that no studies investigating the relationship between energy balance (i.e., energy intake vs. energy expenditure [74]) and burn-out were found, a positive or negative energy balance may also be associated with burn-out. The interaction and co-existence of energy balance-related behaviors determine whether or not a positive or negative energy balance is experienced [75]. As energy imbalances may lead to the development of overweight and obesity (= physical health) [74], one may hypothesize that a similar imbalance may lead to decreased mental health as well. Previous research, in fact, demonstrates that obesity is associated with higher levels of burn-out [76].

Despite the heterogeneity of populations, assessment methods for both risk of burn-out and physical activity, and physical activity interventions, the vast majority of the experimental studies, including 5 RCTs, concluded that physical activity is effective in reducing the risk of burn-out, suggesting a causal link between both. The experimental studies showed a decrease in burn-out risk ranging from 6.9% to 41.3% [49,52,53,54,55], a decrease in emotional exhaustion between 10% and 33.4% [47,48,51,56] and a decrease in cynicism of 33.3% and 4% [48,51]. One experimental study by Freitas et al. [50], showing a relatively low quality score of 0.63, did not find any significant effect of physical activity on risk of burn-out. This may be due to the small sample size (*n* = 21) and the duration of the physical activities performed in this study. The participants had to perform a 10-min workplace physical activity session on weekdays (no information on type or intensity was provided), while the World Health Organization (WHO) [16] recommends that adults should be physically active at a moderate intensity for at least 150 min per week or at a vigorous intensity for at least 75 min per week or an equivalent combination of both moderate and vigorous intensity activity. So, the total duration of the physical activities performed in the study by Freitas et al. [50] (ten minutes per day, for five days a week) was not meeting the above guidelines [77]. On the other hand, the study by Stenlund et al. [54], in which the performed physical activities (60 min, twice a week, at a moderate intensity) also failed to meet the WHO guidelines, did report a significant effect of physical activity on burn-out. It should be mentioned that, despite not meeting the recommendations, total physical activity duration in the latter study was still much higher compared to the study by Freitas et al. [50] (i.e., 120 min versus 50 min per week, respectively). In another experimental study [55], two groups of participants performed high- and low-intensive physical activities, respectively, for 60 min twice a week (=120 min in total). The low intensive group also failed to meet the WHO guidelines. Nevertheless, both groups (high vs. low intensity) had more or less the same effect on burn-out (reductions of 8.1% and 8.2%, respectively) [55]. These findings suggest that lower (than recommended) amounts of physical activity of 120 min per week (at a low to moderate intensity) may already be effective in reducing burn-out risk. Furthermore, the role of intensity may be questioned. It should be mentioned, however, that the physical activities performed in the other experimental studies (showing a positive effect of physical activity on burn-out risk) were in line with the WHO guidelines, as they lasted 20 to 60 min for two to five times per week at a moderate to vigorous intensity [47,48,49,51,53]. The remaining two experimental studies did not give clear information about the duration of the physical activity sessions [52,56]. Further, four experimental studies [47,52,53,56] also conducted follow-up measurements and showed a decrease in risk of burn-out three, six and 12 months after the interventions, indicating long-term effectiveness, even when the physical activity intervention did not remain in place. 

Our results are in line with the systematic review by Naczenski et al. [1] showing strong evidence for the effect of physical activity on reducing (emotional) exhaustion, but limited evidence for the effect on professional efficacy and cynicism. Further, Naczenski et al. [1] concluded that being physically active one or two times per week for four to 18 weeks has promising effects on reducing burn-out symptoms. The present systematic review slightly deviates from this conclusion, showing that positive effects on burn-out risk were achieved when being physically active two to five times per week for 20 to 60 min, for six to 18 weeks, with 18 weeks showing the biggest reduction in burn-out risk (−47.8%) [53]. The latter suggests that the longer the duration of the physical activity intervention, the higher the reduction in burn-out risk. However, due to the large variety in type, intensity, duration and frequency of the performed physical activities in the included studies, comparison of the effectiveness of the individual interventions remains difficult. Further research to unravel the respective effects of type, intensity, duration and frequency is therefore highly recommended. Furthermore, to better understand the relationship between physical activity and burn-out risk, it is also important to investigate the underlying physiological mechanisms. For example, the role of BDNF might be interesting as BDNF-levels increase when physical activity is performed, while on the other hand, BDNF-levels were found to be decreased in people having burn-out [22]. Four experimental studies [49,52,54,56] compared physical activity interventions with other interventions—such as basic care, cognitive interventions and a multimodal rehabilitation program—and did not find physical activity to be more effective compared to the other treatment arms. It should be mentioned that, in one of these studies [52], the aforementioned conclusion was based on the results of six and 12 months after the intervention was completed, possibly causing differences between treatment effects to have been diminished. Another possible reason for these results may be a shared effect between the interventions, resulting in no effect of intervention type [78]. As suggested by Heiden et al. [52], the same (psychosocial) attention was given to all patients in both interventions, suggesting (psychosocial) attention to be such a shared effect. Furthermore, as human interaction was also present in the other experimental studies (e.g., interaction between the researcher or therapist and the participant during the intervention phase), part of the intervention effects might be explained by the same psychosocial component [49,52,54,56]. 

These results might suggest that physical activity may be equally effective compared to other types of interventions, as long as there is a psychosocial component involved. Future research should further unravel the relative importance of physical activity versus other intervention components when aiming at reducing burn-out risk.

Regarding sedentary behavior, only positive relationships with risk of burn-out were reported, indicating that higher levels of sedentary behavior are associated with higher burn-out risk. It should be mentioned, however, that only two observational studies [62,67] were found, making it difficult to draw any firm conclusions. Nevertheless, the quality score of these two studies was high (0.86 and 0.95). Besides, it is important to mention that these studies primarily aimed to assess the relationship between physical activity and risk of burn-out. In both studies, the assessment method of physical activity was the 4-level Saltin Grimby Physical Activity Level Scale [79], of which the lowest level reflects sedentary behavior. This level means that participants were not participating in any leisure-time physical activities or sport activities, which is not in line with the definition of sedentary behavior [25]. Furthermore, no validated objective (e.g., inclinometers, accelerometers) nor subjective (e.g., more detailed context-specific questionnaires) assessment methods for sedentary behavior were used. Despite the fact that sedentary behavior may influence mental health and despite its impact on the risk of the occurrence of common mental disorders such as depression, decreased mood and anxiety, no experimental studies investigating the influence of sedentary behavior on burn-out risk were found. Furthermore, the study by Bernaards et al. [58] concluded that the association between physical activity and burn-out risk was stronger in workers with a sedentary job. This suggests a possible moderating role of sedentary behavior in the physical activity-burn-out risk relationship pathway. So, the interaction between physical activity and sedentary behavior should be further investigated while explaining burn-out risk.

Two observational studies [46,69], with quality scores of 0.80 and 0.91 respectively, found that a healthier diet is related to a lower risk of burn-out. Because only two studies were found and the fact that these two studies were investigating different aspects of dietary behavior, namely fast food consumption and the amount of healthy diets during work days, it is difficult to draw reliable conclusions regarding this relationship. Moreover, these two studies did not use valid and reliable assessment methods to measure both aspects. Alexandrova-Karamanova et al. [46] measured fast food consumption by one single self-constructed question, namely “How many times in a week do you eat fast food?”. In the study by Gorter et al. [69] dietary behavior was measured by asking how many healthy diets participants consumed during workdays, while “healthy diets” was not defined. These methodological shortcomings make it even more difficult to draw firm conclusions. 

It has been shown that diet may influence mental health. A systematic review and meta-analysis by Tolkien et al. [80] for example, concluded that an anti-inflammatory diet may play an important role in preventing or reducing depression risk and symptoms. Moreover, because of the link between burn-out and neurotransmission and the role diet may play herein, it is important to further investigate the link between dietary behavior and burn-out risk by conducting experimental research using valid and reliable assessment methods (e.g., food diaries, 24-hour recalls or food frequency questionnaires).

Because all studies regarding sedentary behavior, dietary behavior and burn-out risk had an observational design, no conclusions about the causal relationship can be made. As hypothesized above, being sedentary may increase the risk of burn-out through different physiological mechanisms, while a burn-out may also cause people to be more sedentary, and so reversed causality is possible. The same may be true for dietary behavior. This shows the need for more experimental studies investigating the causal relationship between sedentary and dietary behavior and burn-out risk. 

There are several limitations to the included studies. A first limitation is the fact that some studies [57,58,69] measured physical activity with only one or two single self-constructed questions. Moreover, some measurement methods for physical activity had methodological shortcomings. The study by Liang et al. [61], for example, measured exercise behavior on a 5-point scale with anchors 1: ‘never’ to 5: ‘many times in a week’, while ‘many times’ was not defined. Future research should use validated questionnaires (e.g., International Physical Activity Questionnaire [81]), and preferably objective measures, such as accelerometers or pedometers. The same can be said for sedentary and dietary behavior. A second limitation is that less than half of the included studies used the Maslach Burnout Inventory (MBI), while the MBI is the gold standard assessment tool for burn-out [82]. All the other assessment tools for burn-out used in the included studies are based on other definitions of burn-out and are mostly measuring one dimension, namely (emotional) exhaustion. It is recommended that future research uses the gold standard assessment tool for measuring burn-out, in order to increase measurement homogeneity across studies. A third limitation is that some studies are mixing up the terms “physical inactivity” and “sedentary behavior”. Two of the included studies [57,63] use a physical activity questionnaire to classify people as active or inactive, and considered inactive people to be sedentary, while literature clearly shows that physical inactivity and sedentary behavior are two different concepts [27,28,83]. A fourth limitation is that the majority of the included studies did not take physiological, psychological and sociological confounders into account, and so conclusions may have to be interpreted with caution. A fifth limitation is that most studies consisted mostly of female participants, which may have influenced the results. Research shows that women generally eat healthier but are less physically active compared to men [84,85,86,87,88,89]. Moreover, a meta-analysis showed that women are more likely to report burn-out [90]. More specifically, women reported to be more emotionally exhausted than men, while men were more depersonalized [90]. Hence, future studies investigating the association between energy balance-related behavior and burn-out should take sex into account. Lastly, some articles were missing some relevant information, such as how physical inactivity was measured, so the authors had to be contacted. Unfortunately, we did not always get a response leaving some queries unanswered. 

There are also a few limitations to the present systematic review. Since non-English written publications were excluded, we may have missed out on important scientific articles in other languages. Additionally, the used quality assessment tool does not distinguish between experimental and observational studies. Experimental studies can be considered higher in quality and so they should receive a higher score in the quality assessment. However, when calculating the mean quality score per study design, a higher mean quality score of 0.87 ± 0.08 for the observational studies was found, compared to a mean quality score of 0.74 ± 0.08 among the experimental studies.

A strength of this systematic review is the fact that this is the first systematic review aiming to include articles of all study designs investigating the relationship between energy balance-related behavior as a whole (i.e., the combination of physical activity, sedentary behavior and dietary behavior) and burn-out risk. As—in the present review—no studies using this holistic approach were found, and because of the hypothesized role these three components may play in reducing or preventing burn-out, further research on this topic is needed.

## 5. Conclusions

This systematic review shows that any type of physical activity, lasting 20 to 60 min and performed two to five times per week for six to 18 weeks, may be effective in reducing the risk of burn-out. The few observational studies linking sedentary and dietary behavior with burn-out risk suggest that engaging in frequent sedentary behavior and eating less healthy are each associated with higher burn-out risk. More high-quality research is needed to unravel the causal relationship between sedentary and dietary behavior and the risk of burn-out. 

## Figures and Tables

**Table 1:** Overview of the included experimental studies.

Author (year) and Country	Design	Participants and Setting	Intervention	Timing of Measurements	Outcome Measure and Measurement Tool	Conclusion
Physical activity						
De Vries et al. [47] The Netherlands	RCT	96 employees with high levels of work-related fatigue: - 19 men, 77 women - Mean age: 45.2 ± 1.6 years	Exercise intervention: 1 h low-intensity running sessions three times a week for 6 consecutive weeks. Wait-list control: participants were offered the opportunity to follow the intervention after the 6 weeks training of the IG.	IG: pre-intervention and post-intervention, 6 and 12 weeks after intervention period. CG: pre-intervention and post-intervention.	Work-related fatigue: five-item ‘exhaustion’-subscale of the Dutch version of the MBI.	Exercise is effective to reduce emotional exhaustion (T0-T1: cohen’s d = −0.62; −21.6%; *p =* 0.04). Small improvements in emotional exhaustion 6 weeks after the end of the intervention. These were maintained at 12 weeks (T0-T2: cohen’s d = −1.03; −33.8%; *p <* 0.01; T0-T3: cohen’s d = −1.06; −32.6%; *p <* 0.01; T1-T2: cohen’s d = −0.3, −15.6%, *p <* 0.05). No significant difference between T1 and T3 and between T2 and T3.
Dreyer et al. [48] New Zealand	RCT	81 staff members at a college: - 25 men, 56 women - Mean age: 42.1 years	The exercise intervention lasted for 10 weeks (4–5 days per week). The exercise intervention: a combination of aerobic exercise (cycling, stair climber, treadmill running) and resistance exercise. Aerobic exercise: participants started at 40–50% of age-adjusted maximum heart rate with 40-min sessions for the first 2 weeks, followed by 50–60% with 30-min sessions during the next 3 weeks, and 70% or higher with 20-min sessions for the last 4 weeks. Resistance exercise: 4–5 sets of 4 exercises with a 30-second recovery interval between sets. The entire circuit had to be completed within 40 min. CG: no intervention.	Pre, post (week immediately after the intervention).	Emotional exhaustion: Psychological Burn-out Questionnaire.	Emotional exhaustion improved significantly after the 10-week exercise intervention (−10%).
Eskilsson et al. [49] weden	RCT	56 patients with exhaustion disorder. All patients were on sick leave: - 4 men, 52 women - Mean age: 41.8 ± 8.2 years	Multimodal rehabilitation program containing components of group-based or individual cognitive behavioral therapy, physical activities and work training coordinated by an interdisciplinary team. 12-week intervention: Aerobic training was performed as group indoor cycling (spinning), 40 min, 3 times per week. Intensity: 70–85% of their maximum age-adjusted heart rate. CG: Multimodal rehabilitation program with no additional training.	Baseline, week 12, week 24.	Burn-out: SMBQ.	No additional improvement in burn-out in the aerobic group compared to controls. Levels of burn-out improved equally in both groups (aerobic training group: T1-T2: −17.5%, no *p*-value mentioned).
Freitas et al. [50] razil	Pre-experiment	21 nursing professionals (from the Barretos Cancer Hospital): - 1 man, 20 women - Mean age: 37.4 ± 9.1 years - employed for 1 year or more	The compensatory workplace PA was conducted 5 days/week, lasting 10 min, during 3 consecutive months. No CG.	Pre, post	Burn-out: MBI	No significant difference in the three dimensions of burn-out between pre- and post-intervention.
Gerber et al. [51] wiss	Pre-experiment	Employees with high levels of work-related burn-out: - 12 men - Mean age: 45.8 ± 6.8 years	12-week exercise-training program, 2–3 times a week, 60 min: aerobic exercise program based on the exercise prescription guidelines of the American College of Sports Medicine. 60–75% of their maximum heart rate. No CG.	Pre, post (3 days after the 12-week intervention).	Burn-out: German version of the MBI.	Burn-out symptoms were significantly reduced after the 12-week aerobic exercise program. Emotional exhaustion and depersonalization were reduced. No significant changes were found for personal accomplishment (Emotional exhaustion: T1-T2: cohen’s d = 1.84; −33.4%; *p <* 0.001; depersonalization: T1-T2: cohen’s d = 1.35; −33.5%: *p <* 0.001; personal accomplishment: T1-T2: no significant difference).
Heiden et al. [52] Sweden	RCT	75 patients being on sick leave for at least 50% of the time for 1 month to 2 years due to stress-related illnesses: - 15 men, 60 women - Mean age: 44.0 ± 9.0 years	PA programme: 2 exercise sessions per week for 10 weeks. Session 1: a rehabilitation program with low-intensity exercises in a warm water pool. Session 2: the participants chose an exercise (e.g., strength training, swimming, aerobics or walking). During the intervention, each participant kept a diary of their physical exercise. The cognitive behavioral training programme focused on cognitive restructuring to improve self-care behavior and social support. CG: usual care provided by the Swedish social insurance system during the course of the study. Participants were promised treatment after the study was completed.	Pre, post, at 6 and at 12 months after the intervention.	Burn-out: SMBQ.	Participants rated lower levels of burn-out after the intervention period (F=10.0; *p =* 0.002). At the 6-month follow up assessment, patients’ ratings of burn-out continued to improve. At 12 months after the intervention, similar results were found. Little difference in the effect of cognitive behavioral training and PA, compared with usual care, was found (PA intervention: pre-post: −12.1%; cognitive behavioral training: pre-post: −12.9%; usual care pre-post: +2.9%, no *p*-values mentioned)
Lindegard et al. [53] Sweden	Pre-experiment	69 patients with exhaustion disorder on sick leave for less than 6 months: - 24 men, 69 women - Mean age: 42.6 ± 1.4 years - Only physically inactive patients at baseline were included.	Multimodel treatment for 12 months and a special focus was placed on PA counselling: an 8-week group stress management program. All patients were given background information on the causes and consequences of chronic stress during a 2-h lecture. They were visiting every 4–6 weeks a physician and the program consisted of a 2 h lecture about stress-related mental disorders and the consequences on the individual and organisational level. The participants were also given comprehensive information of the effects of regular PA on stress-related exhaustion, opportunity to self-select their participation in an 18-week coached group exercise-program. Exercise program: Nordic walking for 1 h and a light strength-training program performed at the clinic once a week. No CG.	Pre, at 6 months, at 12 months, at 18 months.	Burn-out: SMBQ. PA activity: at baseline: with the SGPALS, at follow-up: “How often did you exercise during the last 3 months?” and “How hard did you normally exercise during the last 3 months?” and “How many minutes did you engage in activity?”	Mild and strong compliers reported significantly lower burn-out at the 18-month follow-up than the non-complying group (pre-18 months: non-compliers: −27.7%; mild-compliers: −47.8%; strong compliers: −41.3%; *p <* 0.017).
Stenlund et al. [54] Sweden	RCT	82 patients with burn-out: - 14 men, 68 women - Mean age: 44.3 ± 9.1 years	IG: Qigong twice a week (1-h sessions) for 12 weeks. The Qigong program: warm-up movements, basic movements to affect body awareness, balance and coordination, breathing and muscular tension, and relaxation and mindfulness meditation. The IG also took part in basic care at the Stress Clinic. The CG took part in basic care at the Stress Clinic.	Pre, at week 4 and week 8 of the intervention and after the intervention.	Burn-out: SMBQ.	Both groups improved significantly with reduced levels of burn-out. No additional effects of Qigong on recovery in burn-out patients compared to basic care for patients with burn-out (IG: pre- post: −6.9%; *p <* 0.001; CG: pre-post: −17.4%; *p <* 0.001).
Tsai et al. [55] China	Quasi-experiment	89 banking and insurance workers: - Low intensity group: n = 30, 11 men, 19 women, mean age: 34.8 ± 7.0 years - High intensity: n = 29, 3 men, 27 women, mean age: 41.0 ± 7.2 years - CG: n = 29, 10 men, 19 women, mean age: 33.3 ± 9.4 years,	12-week exercise program: gymnastics (15 min); aerobic exercise (30 min) and stretching (15 min). Low-intensity group: participants were assigned to attend 1 exercise session per week. High-intensity group: participants were assigned to attend 2 exercise sessions per week. Participants planned and carried out exercise regimes on their own.	Pre, post	Work-related burn-out: CBI.	The exercise program improved work-related burn-out. (High-intensity exercise: pre- post: −8.1%; low-intensity exercise: pre-post: −8.2%; no *p*-values mentioned).
Van Rhenen et al. [56] The Netherlands	Randomised clinical trial	75 employees working in a telecommunications company: - Sex distribution not mentioned. - Age: between 18.0 and 63.0 years	Condition 1: Physical intervention: to provide awareness and introduction of physical and relaxation exercises in daily work activities. The level and intensity of the exercises were modified in such a way that it met the physical capability of each individual. The sessions took place during working hrs. Four sessions, each lasting for 1 h, were given over a period of 8 weeks. Every session consisted of 4 main parts: introduction, warming-up and physical exercise, relaxation exercise and an assignment. Condition 2: Stressed cognitive intervention: to restructure irrational beliefs. Participants were randomly assigned to 1 of the 2 conditions. No CG.	Pre, 10 weeks and 6 months after the training period.	Burn-out: UBOS, the Dutch version of the MBI-General Survey.	Both interventions had a positive effect on exhaustion and cynicism in both the short and long term (physical intervention: exhaustion: pre-post: −10%; *p =* 0.04; cynicism: pre-post: −4%; *p =* 0.04: cognitive intervention: exhaustion: pre-post: −4%: *p =* 0.04; cynicism: pre-post: −9.3%; *p =* 0.04).

Abbreviations: Maslach Burn-out Inventory (MBI), Shirom-Melamed Burn-out Questionnaire (SMBQ, Copenhagen Burn-out Inventory (CBI), Utrechtse Burn-out Schaal (UBOS), Physical Activity (PA) Intervention group (IG), Control group (CG), Randomised Controlled Trial (RCT).

**Table 2:** Overview of the included observational studies.

Author (year) and Country	Design	Participants and Setting	Outcome Measure and Measurement Tools	Conclusion
Physical activity				
Ahola et al. [57] Finland	Cross-sectional	3264 participants: - 1645 men, 1619 women - Mean age: 44.5 years	Burn-out: MBI - General Survey. Leisure-time PA: questionnaire. Participants who reported spending at least 4 h of their weekly leisure-time in physical activities = “active”, and those who reported spending most of their leisure-time in non-physical activities = “passive”.	Burn-out syndrome was related to low PA: OR, 1.21; 95% CI, 1.12–1.30). Exhaustion (OR, 1.23; 95% CI, 1.12–1.32), cynicism (OR, 1.10; 95% CI, 1.01–1.19) and a lack of professional efficacy (OR, 1.13; 95% CI, 1.06–1.22) were associated with low PA levels.
Bernaards et al. [58] The Netherlands	Longitudinal with 4 time points: baseline measurements between 1994 and 1995, and follow-up measurements in 1996, 1997 and 1998.	1747 workers from 34 companies (blue-and white-collar jobs and caring professions) - Sex distribution and age were not mentioned. - Participants had to be employed in their current job for at least 1 year and work at least 24 h per week.	Emotional exhaustion: it was assessed with one of the three subscales from an adapted Dutch version of the MBI. Strenuous PA: “How often within the past four months did you participate in strenuous sports activities or strenuous physical activities that lasted long enough to become sweaty?” The amount of sedentary work: participants reporting sitting during the largest part of the working day = workers with a sedentary job, participants not reporting sitting during the largest part of the working day = worker with a non-sedentary job.	All workers who engaged in strenuous PA at a frequency of one to twice a week were at a significant lower risk of emotional exhaustion than workers who engaged in strenuous PA less than once a month. This association was stronger in workers with a sedentary job. PA at a frequency of once to twice a week was significantly associated with a reduced risk of future emotional exhaustion, this was not the case for PA at a higher frequency. (1–2x per week: non-sedentary job: OR, 0.70; 95% CI, 0.51–0.97; sedentary job: OR, 0.48; 95% CI, 0.30–0.76)
Carson et al. [59] USA	Cross-sectional	189 full-time childcare teachers (African, American, Caucasian-American): - 1 man, 188 women - Mean age: 33.6 ± 12.4 years	Emotional exhaustion: the nine-item emotional exhaustion subscale from the MBI-Educators Survey. Self-reported PA behavior: Baecke’s Habitual Physical Activity Questionnaire: 16 items delineated into 3 distinct indices: work index, sport-related index and leisure-time index.	Work related PA (r = −0.3, *p <* 0.01) and leisure-time PA (r = −0.19, *p <* 0.05) were negatively correlated with emotional exhaustion.
de Vries et al. [60] The Netherlands	Longitudinal with 2 time points: measurements in 2008 and 2009.	2275 full-time employees: - 75.3% men → mean age: 45.8 ± 10.0 years - 24.7% women → mean age: 39.9 ±11.4 years) - The participants primarily worked in the area of business services, public administration, industry, and education (no physically demanding jobs) - Mean working hrs per week: 38.4 ± 3.1 - Mean working days per week: 4.9 ± 0.5	Work-related fatigue: five-item ‘exhaustion’-subscale of the Dutch version of the MBI. PA: questionnaire based on international standards for PA: “On how many days a week are you normally physically active during at least 30 min a day during your work and free time together (only count PA that is equally demanding as brisk walking or biking. Activities shorter than 10 min do not count)?”	It was found that an increase in PA is associated with a decrease in work-related fatigue over time (β = −0.05, p <0.05). Cross-sectionally, work-related fatigue is negatively correlated with PA at T1 and T2 (T1: r = −0.08, *p <* 0.01; T2: r = −0.08, *p <* 0.001)
Liang et al. [61] Taiwan	Longitudinal	197 full-time employees in five manufacturing industries: - 163 men, 34 women - Age: not mentioned	Burn-out: CBI. Exercise behavior: 5 questions on a 5-point scale: 1 = never and 5 = many times in a week. Example questions: “How many times in a week do you take part in sports that include aerobic exercise (e.g., basketball and running)?” and “How many times in a week do you engage in aerobic exercise for at least 30 min?”	Work-related burn-out was negatively correlated with exercise behavior (r = −0.22, *p <* 0.01).
Lindwall et al. [62] Sweden	Longitudinal with 5 time points: baseline, in 2004, in 2006, in 2008 and in 2010.	3717 health care workers: - Sex distribution not mentioned. - Mean age: 46.9 ± 10.0 years - Criteria for inclusion: at least 1 full year of employment and working at least halftime.	Burn-out: SMBQ. PA: adapted version of the 4-level Saltin Grimby Physical Activity Level Scale (reporting PA in the last 3 months). This scale makes a distinction between participants who are mostly sedentary, who engage in light PA for at least 2 h a week, who report at least 2 h per week of moderate PA or who engage in vigorous activity at least 5 h per week on several occasions.	More PA is associated with fewer symptoms of burn-out at a cross-sectional level at baseline (r = −0.4, *p <* 0.01). Individuals who became more active compared to others across the 6 years also showed a larger decrease in symptoms of burn-out (r = −0.79, *p <* 0.01).
Moueleu Ngalagou et al. [63] Cameroon	Cross-sectional	303 teaching staff members (lecturers, senior lecturers, professors): - 209 men, 94 women - Mean age: 43.0 ± 7.0 years	Burn-out: MBI. PA and sport practice: Ricci-Gagnon Questionnaire was used to assess the level of physical activities and sport practice.	Individuals reporting LPA or MVPA were significantly less likely to be classified as having elevated scores on burn-out compared to those who were inactive (LPA: OR, 0.13; 95% CI, 0.12–0.73); MVPA: OR, 0.14: 95% CI, 0.05–0.35).
Hu et al. [64] Taiwan	Cross-sectional	1560 full-time employees: - Mean age: 45.4 ± 8.9 years	Burn-out: CBI. It was not mentioned how physical inactivity was measured.	A positive correlation between physical inactivity and being in the upper tertile (range 37.5 to 100) of burn-out was found (lower tertile: 37.8% physically inactive, middle tertile: 38.4% physically inactive, upper tertile: 57.6% physically inactive; *p* < 0.01) (Upper vs. lower tertile: OR, 1.78; 95% CI, 1.33–2.37; *p* < 0.01).
Peterson et al. [65] Sweden	Cross-sectional	3719 employees (physicians, nurses, nursing assistants, social workers, occupational therapists, physiotherapists, psychologists, dental nurses, hygienists, dentists, administrators, teachers and technicians) in a Swedish Country Council: - 18% men, 82% women - Age range: between 22 and 66 years	Burn-out: OBI measuring 2 dimensions: exhaustion and disengagement. PA: frequency of physical exercise was assessed on a five-point scale scoring from ‘never’ to ‘3 times per week or more’. Exercising 2 times per week or more was classified as high, and ‘never’ and ‘irregularly’ was categorized as low PA.	Physical exercise played a minor role in discriminating between burn-out and non-burn-out groups: Emotional exhaustion and exercise: r = 0.12 (no *p*-value mentioned). Disengagement and exercise: r = 0.04 (no *p*-value mentioned).
Sane et al. [66] Iran	Cross-sectional	81 teachers of Danegaz University.	Burn-out: MBI. PA: Baecke’s physical activity questionnaire	There is an inverse correlation between PA and burn-out (r = −0.4, *p =* 0.001).
Toker et al. [21] Israel	Longitudinal with 3 time points between 2003 and 2009.	1632 employees (working in high and low technology, teaching or academia, administration, sales and services, blue collar, health care): - 70% men, 30% women - Mean age: 46.6 ± 8.7 years - Working for minimum 50% (32% managerial position)	Burn-out: SMBQ. PA: based on patients’ self-reports. Consistent with the American College of Sports Medicine and the American Heart Association guidelines, they were asked how many days per week and how many minutes each session they engaged over the past month in strenuous PA (activity that increases the heart rate and brings on a sweat) during their leisure time.	PA and burn-out are negatively correlated (job burn-out – PA T1: r = −0.10, *p <* 0.01; T2: r = −0.11, *p <* 0.01).
Physical activity and sedentary behavior				
Jonsdottir et al. [67] Sweden	Longitudinal with 2 year follow-up (data was collected in 2004 and 2006)	3114 participants (health care workers and workers at the social insurance offices): - 420 men, 2694 women - Mean age: 49.0 ± 9.9 years - Only employees with at least 1 year employment and working at least 50% of a full-time equivalent.	Burn-out: SMBQ PA and sedentary behavior: adapted version of the 4-level Saltin Grimby Physical Activity Level Scale (reporting PA in the last 3 months). This scale makes a distinction between participants who are mostly sedentary, who engage in light PA for at least 2 h a week, who report at least 2 h per week of moderate PA or who engage in vigorous activity at least 5 h per week on several occasions.	Participation in LPA or MVPA was associated with lower reports of high burn-out levels (LPA: Prevalence Ratio, 0.61; 95% CI, 0.51–0.74; MVPA: Prevalence Ratio, 0.40; 95% CI 0.32–0.50). Individuals reporting LPA and MVPA at baseline were less likely to report burn-out at the follow-up compared to those reporting sedentary activity (LPA: PR, 0.59; 95% CI, 0.41–0.85; MVPA: PR, 0.43; 95% CI, 0.28–0.64).
Lindwall et al. [68] Sweden	Cross-sectional	177 employees (health care workers and workers at the social insurance offices): - 87 men, 90 women - Mean age: 39.1 ± 8.1 years - Only employees with at least 1 year employment and working at least 50% of a full-time equivalent.	Burn-out: SMBQ. PA: adapted version of the 4-level Saltin Grimby Physical Activity Level Scale (reporting PA in the last 3 months). This scale makes a distinction between participants who are mostly sedentary, who engage in light PA for at least 2 h a week, who report at least 2 h per week of moderate PA or who engage in vigorous activity at least 5 h per week on several occasions.	Individuals reporting LPA and MVPA were less likely to be classified as having elevated scores on burn-out compared to those who were sedentary (LPA: OR, 0.30; 95% CI, 0.12–0.73; MVPA: OR, 0.14; 95% CI, 0.05–0.35). No differences were found between the LPA and MVPA groups in terms of burn-out.
Physical activity and dietary behavior				
Alexandrova-Karamanova [46] Greece, Portugal, Bulgaria, Romania, Turkey, Croatia and Macedonia	Cross-sectional	2623 health professionals working in university hospitals in Greece, Portugal, Bulgaria, Romania, Turkey, Croatia and Macedonia: - 24.5% men, 75.5% women - Mean age: 38.7 ± 10.2 years - 627 medical doctors, 1431 nurses, 565 residents	Burn-out: MBI-Human services survey PA and dietary behavior: Health Behaviors Questionnaire: PA and healthy eating were both assessed through a single item: “How many times do you exercise per week?” and “How many times in a week do you eat fast food?”	More frequent fast food consumption was significantly associated with higher emotional exhaustion and higher depersonalization (emotional exhaustion: β=0.14; *p <* 0.001; depersonalization: β=0.16; *p <* 0.001) and less frequent exercise (emotional exhaustion: β=−0.17; *p <* 0.001; depersonalization: β=−0.12; *p <* 0.001.
Gorter et al. [69] The Netherlands	Cross-sectional	709 dentists: - 594 men, 114 women - Mean age: 43.0 years (range: 21 – 62 years)	Burn-out: Dutch version of the MBI. PA and dietary behavior: Health behavior: measured by 7 self-constructed items. An example of an item is: “To your opinion, do you eat healthy during work days?”	The high-risk group has a more unhealthy lifestyle, meaning that they perform less physical exercise and they consume less healthy diets during work days compared to the low-risk group (sporting/physical exercise: high-risk group: 28% several times a week; *p <* 0.005; Healthy diet at working days: high-risk group: 29% every day; *p <* 0.005).

Abbreviations: Maslach Burn-out Inventory (MBI), Shirom-Melamed Burn-out Questionnaire (SMBQ), The Oldenburg Burn-out Inventory (OBI), Copenhagen Burn-out Inventory (CBI), Physical Activity (PA) Low Physical Activity (LPA), Moderate-to-vigorous Physical activity (MVPA), Intervention Group (IG), Control Group (CG), Randomised Controlled Trial (RCT).

**Table 3:** Quality assessment of the included studies.

	Research Question	Study Design	Method	Subject	Allocation	Blinding of Investigators	Blinding of Subjects	Outcome	Sample Size	Analytic Methods	Estimate of Variance	Confounding	Results	Conclusions	Summary Score (/1)
Experiment studies															
Physical activity															
de Vries et al. [47]	2	2	1	2	2	0	0	2	2	2	2	2	2	2	0.82
Dreyer et al. [48]	2	1	1	2	1	0	0	1	1	2	2	0	2	2	0.61
Eskilsson et al. [49]	2	2	1	2	1	0	N/A	2	1	2	2	2	2	2	0.81
Freitas et al. [50]	2	1	1	2	N/A	0	N/A	1	0	2	2	0	2	2	0.63
Gerber et al. [51]	1	1	1	2	N/A	0	N/A	2	2	2	2	2	2	2	0.79
Heiden et al. [52]	1	2	1	2	1	0	0	1	1	2	2	2	2	2	0.68
Lindegard et al. [53]	2	1	1	2	N/A	0	N/A	2	1	2	2	2	2	2	0.79
Stenlund et al. [54]	2	2	1	2	2	0	0	2	1	2	2	2	2	2	0.79
Tsai et al. [55]	2	2	1	2	0	0	0	2	2	2	2	2	2	2	0.75
Van Rhenen et al. [56]	1	2	1	2	1	0	0	2	1	2	2	2	2	2	0.71
Observational studies															
Physical activity															
Ahola et al. [57]	2	1	1	2	N/A	N/A	N/A	2	2	2	2	N/A	2	2	0.90
Bernaards et al. [58]	2	2	1	1	N/A	N/A	N/A	2	1	2	2	2	2	2	0.86
Carson et al. [59]	2	1	1	2	N/A	N/A	N/A	2	2	1	2	N/A	2	2	0.85
de Vries et al. [60]	2	2	1	2	N/A	N/A	N/A	2	2	2	2	2	2	2	0.95
Hu et al. [64]	2	2	1	2	N/A	N/A	N/A	1	1	2	2	N/A	2	2	0.85
Liang et al. [61]	2	1	1	2	N/A	N/A	N/A	2	1	2	2	1	2	2	0.82
Lindwall et al. [62]	1	2	1	2	N/A	N/A	N/A	2	1	2	2	2	2	2	0.86
Moueleu Ngalagou et al. [63]	2	2	1	2	N/A	N/A	N/A	2	2	2	2	N/A	2	2	0.95
Peterson et al. [65]	2	2	1	1	N/A	N/A	N/A	2	2	2	2	N/A	2	2	0.9
Sane et al. [66]	1	1	1	0	N/A	N/A	N/A	1	1	2	2	N/A	2	2	0.65
Toker et al. [21]	2	2	1	2	N/A	N/A	N/A	2	1	2	2	2	2	2	0.91
Physical activity and sedentary behavior															
Jonsdottir et al. [67]	2	2	1	2	N/A	N/A	N/A	2	2	2	2	2	2	2	0.95
Lindwall et al. [68]	2	2	1	2	N/A	N/A	N/A	2	1	2	2	2	2	2	0.91
Physical activity and dietary behavior															
Alexandrova- Karamanova et al. [46]	2	2	1	2	N/A	N/A	N/A	1	2	2	2	2	2	2	0.91
Gorter et al. [69]	1	1	1	1	N/A	N/A	N/A	2	2	2	2	N/A	2	2	0.80

## References

[1] Naczenski L.M., De Vries J.D., Van Hooff M.L.M., Kompier M.A.J. (2017). Systematic review of the association between physical activity and burnout. J. Occup. Heal..

[2] Leka S., Jain A., World Health Organization (2010). Health Impact of Psychosocial Hazards at Work: An Overview.

[3] Ferreira A.I., Martinez L.F. (2012). Presenteeism and burnout among teachers in public and private Portuguese elementary schools. Int. J. Hum. Resour. Manag..

[4] Harvey E., Burns J. (1994). Staff burnout and absenteeism through service transition: From hospital to hostel. Ment. Handicap Res..

[5] Wallace J.E., Lemaire J.B., Ghali W.A. (2009). Physician wellness: A missing quality indicator. Lancet.

[6] Ochentel O., Humphrey C., Pfeifer K. (2018). Efficacy of Exercise Therapy in Persons with Burnout. A Systematic Review and Meta-Analysis. J. Sports Sci. Med..

[7] Rowe D.S. The Stress Burden: Strategies for Management. https://www.thefreelibrary.com/The+stress+burden%3a+strategies+for+management.-a0288874874.

[8] EU-OSHA (2014). Calculating the Cost of Work-Related Stress and Psychosocial Risks.

[9] Maslach C., Schaufeli W.B., Leiter M.P. (2001). Job Burnout. Annu. Rev. Psychol..

[10] Schaufeli W.B., Leiter M.P., Maslach C. (2009). Burnout: 35 years of research and practice. Career Dev. Int..

[11] Guan S., Xiaerfuding X., Ning L., Lian Y., Jiang Y., Liu J., Ng T.B. (2017). Effect of Job Strain on Job Burnout, Mental Fatigue and Chronic Diseases among Civil Servants in the Xinjiang Uygur Autonomous Region of China. Int. J. Environ. Res. Public Heal..

[12] Awa W.L., Plaumann M., Walter U. (2010). Burnout prevention: A review of intervention programs. Patient Educ. Couns..

[13] Melnyk B.M., Jacobson D., Kelly S., O’Haver J., Small L., Mays M.Z. (2009). Improving the Mental Health, Healthy Lifestyle Choices, and Physical Health of Hispanic Adolescents: A Randomized Controlled Pilot Study. J. Sch. Heal..

[14] Jacka F.N., Mykletun A., Berk M. (2012). Moving towards a population health approach to the primary prevention of common mental disorders. BMC Med..

[15] Lucas M., Mekary R., Pan A., Mirzaei F., O’Reilly É.J., Willett W.C., Koenen K., Okereke O.I., Ascherio A. (2011). Relation Between Clinical Depression Risk and Physical Activity and Time Spent Watching Television in Older Women: A 10-Year Prospective Follow-up Study. Am. J. Epidemiol..

[16] WHO (2010). Global Recommendations on Physical Activity for Health.

[17] Ainslie P.N., Cotter J.D., George K.P., Lucas S., Murrell C., Shave R., Thomas K.N., Williams M.J.A., Atkinson G. (2008). Elevation in cerebral blood flow velocity with aerobic fitness throughout healthy human ageing. J. Physiol..

[18] Colcombe S.J., Kramer A.F., Erickson K.I., Scalf P., McAuley E., Cohen N.J., Webb A., Jerome G.J., Marquez D.X., Elavsky S. (2004). Cardiovascular fitness, cortical plasticity, and aging. Proc. Natl. Acad. Sci. USA.

[19] Erickson K.I., Voss M.W., Prakash R.S., Basak C., Szabo A., Chaddock L., Kim J.S., Heo S., Alves H., White S.M. (2011). Exercise training increases size of hippocampus and improves memory. Proc. Natl. Acad. Sci. USA.

[20] Sonnentag S. (2012). Psychological Detachment from Work During Leisure Time: The Benefits of Mentally Disengaging from Work.

[21] Toker S., Biron M. (2012). Job burnout and depression: Unraveling their temporal relationship and considering the role of physical activity. J. Appl. Psychol..

[22] He S., Zhang Y., Zhan J., Wang C., Du X., Yin G., Cao B., Ning Y., Soares J., Zhang X. (2017). Burnout and cognitive impairment: Associated with serum BDNF in a Chinese Han population. Psychoneuroendocrinology.

[23] Schwarzer R., Hallum S. (2008). Perceived Teacher Self-Efficacy as a Predictor of Job Stress and Burnout: Mediation Analyses. Appl. Psychol..

[24] Westman M., Etzion D. (2001). The impact of vacation and job stress on burnout and absenteeism. Psychol. Heal..

[25] Barnes J., Behrens T.K., Benden M.E., Biddle S., Bond D., Brassard P. (2012). Letter to the editor: Standardized use of the terms “sedentary” and “sedentary behaviours”. Appl. Physiol. Nutr. Metab..

[26] Mansoubi M., Pearson N., Biddle S.J., Clemes S. (2014). The relationship between sedentary behaviour and physical activity in adults: A systematic review. Prev. Med..

[27] van der Ploeg H.P., Hillsdon M. (2017). Is sedentary behaviour just physical inactivity by another name?. Int. J. Behav. Nutr. Phys. Act..

[28] Panahi S., Tremblay A. (2018). Sedentariness and Health: Is Sedentary Behavior More Than Just Physical Inactivity?. Front. Public Health.

[29] De Rezende L.F.M., Lopes M.R., Rey-Lopez J.P., Matsudo V.K.R., Luiz O.D.C. (2014). Sedentary Behavior and Health Outcomes: An Overview of Systematic Reviews. PLoS ONE.

[30] Hoang T.D., Reis J., Zhu N., Jacobs D.R., Launer L.J., Whitmer R.A., Sidney S., Yaffe K. (2016). Effect of Early Adult Patterns of Physical Activity and Television Viewing on Midlife Cognitive Function. JAMA Psychiatry.

[31] Kesse-Guyot E., Charreire H., Andreeva V.A., Touvier M., Hercberg S., Galan P., Oppert J.M. (2012). Cross-sectional and longitudinal associations of different sedentary behaviors with cognitive performance in older adults. PLoS ONE.

[32] Engeroff T., Fuzeki E., Vogt L., Fleckenstein J., Schwarz S., Matura S., Pilatus U., Deichmann R., Hellweg R., Pantel J. (2018). Is Objectively Assessed Sedentary Behavior, Physical Activity and Cardiorespiratory Fitness Linked to Brain Plasticity Outcomes in Old Age?. Neuroscience.

[33] Teychenne M., Ball K., Salmon J. (2010). Sedentary Behavior and Depression Among Adults: A Review. Int. J. Behav. Med..

[34] Endrighi R., Steptoe A., Hamer M. (2016). The effect of experimentally induced sedentariness on mood and psychobiological responses to mental stress. Br. J. Psychiatry.

[35] Tops M., Boksem M.A., Wijers A.A., van Duinen H., Den Boer J.A., Meijman T.F., Korf J. (2007). The psychobiology of burnout: Are there two different syndromes?. Neuropsychobiology.

[36] Yao Y., Zhao S., Zhang Y., Tang L., An Z., Lu L., Yao S. (2018). Job-related burnout is associated with brain neurotransmitter levels in Chinese medical workers: A cross-sectional study. J. Int. Med. Res..

[37] Marriott B.M. (1994). Food Components to Enhance Performance: An Evaluation of Potential Performance-Enhancing Food Components for Operational Rations.

[38] Dye L., Lluch A., Blundell J.E. (2000). Macronutrients and mental performance. Nutrition.

[39] Kaplan R.J., Greenwood C.E., Winocur G., Wolever T.M. (2000). Cognitive performance is associated with glucose regulation in healthy elderly persons and can be enhanced with glucose and dietary carbohydrates. Am. J. Clin. Nutr..

[40] Kennedy D.O., Scholey A. (2000). Glucose administration, heart rate and cognitive performance: Effects of increasing mental effort. Psychopharmacology.

[41] Chung Y.C., Park C.H., Kwon H.K., Park Y.M., Kim Y.S., Doo J.K., Shin D.H., Jung E.S., Oh M.R., Chae S.W. (2012). Improved cognitive performance following supplementation with a mixed-grain diet in high school students: A randomized controlled trial. Nutrition.

[42] Fontani G., Corradeschi F., Felici A., Alfatti F., Bugarini R., Fiaschi A.I., Cerretani D., Montorfano G., Rizzo A.M., Berra B. (2005). Blood profiles, body fat and mood state in healthy subjects on different diets supplemented with Omega-3 polyunsaturated fatty acids. Eur. J. Clin. Investig..

[43] Moher D., Liberati A., Tetzlaff J., Altman D.G., PRISMA Group (2009). Preferred Reporting Items for Systematic Reviews and Meta-Analyses: The PRISMA Statement. Ann. Intern. Med..

[44] da Costa Santos C.M., de Mattos Pimenta C.A., Nobre M.R. (2017). The PICO strategy for the research question construction and evidence search. Rev. Latino-Americana de Enferm..

[45] Kmet L.M., Cook L.S., Lee R.C. (2004). Standard Quality Assessment Criteria for Evaluating Primary Research Papers from a Variety of Fields. HTA Initiative.

[46] Alexandrova-Karamanova A., Todorova I., Montgomery A., Panagopoulou E., Costa P., Băban A., Davas A., Milošević M., Mijakoski D. (2016). Burnout and health behaviors in health professionals from seven European countries. Int. Arch. Occup. Environ. Heal..

[47] De Vries J.D., Van Hooff M.L., Guerts S.A., Kompier M.A., Jd D.V., Mlm V.H., Sae G., Maj K. (2017). Exercise to reduce work-related fatigue among employees: A randomized controlled trial. Scand. J. Work. Environ. Heal..

[48] Dreyer S., Dreyer L., Rankin D. (2012). Effects of a 10-week High-Intensity Exercise Intervention on College Staff with Psychological Burnout and Multiple Risk Facts. J. Res..

[49] Eskilsson T., Järvholm L.S., Gavelin H.M., Neely A.S., Boraxbekk C.-J. (2017). Aerobic training for improved memory in patients with stress-related exhaustion: A randomized controlled trial. BMC Psychiatry.

[50] Freitas A.R., Carneseca E.C., Paiva C.E., Paiva B.S.R. (2014). Impact of a physical activity program on the anxiety, depression, occupational stress and burnout syndrome of nursing professionals1. Rev. Latino-Americana de Enferm..

[51] Gerber M., Brand S., Elliot C., Holsboer-Trachsler E., Pühse U., Beck J. (2013). Aerobic exercise training and burnout: A pilot study with male participants suffering from burnout. BMC Res. Notes.

[52] Heiden M., Lyskov E., Nakata M., Sahlin K., Sahlin T., Barnekow-Bergkvist M. (2007). Evaluation of cognitive behavioural training and physical activity for patients with stress-related illnesses: A randomized controlled study. Acta Derm. Venereol..

[53] Lindegård A., Jonsdottir I.H., Börjesson M., Lindwall M., Gerber M. (2015). Changes in mental health in compliers and non-compliers with physical activity recommendations in patients with stress-related exhaustion. BMC Psychiatry.

[54] Stenlund T., Birgander L.S., Lindahl B., Nilsson L., Ahlgren C. (2009). Effects of Qigong in patients with burnout: A randomized controlled trial. J. Rehabil. Med..

[55] Tsai H.H., Yeh C.Y., Su C.T., Chen C.J., Peng S.M., Chen R.Y. (2013). The effects of exercise program on burnout and metabolic syndrome components in banking and insurance workers. Ind. Heal..

[56] Van Rhenen W., Blonk R.W.B., Van Der Klink J.J.L., Van Dijk F.J.H., Schaufeli W.B. (2005). The effect of a cognitive and a physical stress-reducing programme on psychological complaints. Int. Arch. Occup. Environ. Heal..

[57] Ahola K., Pulkki-Raback L., Kouvonen A., Rossi H., Aromaa A., Lonnqvist J. (2012). Burnout and behavior-related health risk factors: Results from the population-based Finnish Health 2000 study. J. Occup. Environ. Med..

[58] Bernaards C.M., Jans M.P., van den Heuvel S.G., Hendriksen I.J., Houtman I.L., Bongers P.M. (2006). Can strenuous leisure time physical activity prevent psychological complaints in a working population?. Occup. Environ. Med..

[59] Carson R.L., Baumgartner J.J., Matthews R.A., Tsouloupas C.N. (2010). Emotional exhaustion, absenteeism, and turnover intentions in childcare teachers: Examining the impact of physical activity behaviors. J. Health Psychol..

[60] de Vries J.D., Claessens B.J.C., van Hooff M.L.M., Geurts S.A.E., van den Bossche S.N.J., Kompier M.A.J. (2016). Disentangling longitudinal relations between physical activity, work-related fatigue, and task demands. Int. Arch. Occup. Environ. Health.

[61] Liang H.-L., Kao Y.-T., Lin C.-C. (2013). Moderating effect of regulatory focus on burnout and exercise behavior. Percept. Mot. Skills.

[62] Lindwall M., Gerber M., Jonsdottir I.H., Börjesson M., Ahlborg G. (2014). The relationships of change in physical activity with change in depression, anxiety, and burnout: A longitudinal study of Swedish healthcare workers. Health Psychol..

[63] Moueleu Ngalagou P.T., Assomo-Ndemba P.B., Owona Manga L.J., Owoundi Ebolo H., Ayina Ayina C.N., Lobe Tanga M.Y., Guessogo W.R., Mekoulou Ndongo J., Temfemo A., Mandengue S.H. (2018). Burnout syndrome and associated factors among university teaching staff in Cameroon: Effect of the practice of sport and physical activities and leisures. Encephale.

[64] Hu N.-C., Chen J.-D., Cheng T.-J. (2016). The associations between long working hours, physical inactivity, and burnout. J. Occup. Environ. Med..

[65] Peterson U., Demerouti E., Bergstrom G., Samuelsson M., Asberg M., Nygren A. (2008). Burnout and physical and mental health among Swedish healthcare workers. J. Adv. Nurs..

[66] Sane M.A., Devin H.F., Jafari R., Zohoorian Z., Baskan G.A., Ozdamli F., Kanbul S., Ozcan D. (2012). Relationship between physical activity and it’s components with burnout in academic members of Daregaz Universities. 4th World Conference on Educational Sciences.

[67] Jonsdottir I.H., Rödjer L., Hadzibajramovic E., Börjesson M., Ahlborg G. (2010). A prospective study of leisure-time physical activity and mental health in Swedish health care workers and social insurance officers. Prev. Med..

[68] Lindwall M., Ljung T., Hadžibajramović E., Jonsdottir I.H. (2012). Self-reported physical activity and aerobic fitness are differently related to mental health. Ment. Health Phys. Act..

[69] Gorter R.C., Eijkman M.A.J., Hoogstraten J. (2000). Burnout and health among Dutch dentists. Eur. J. Oral Sci..

[70] Carson N.E., Blake C.E., Saunders R. (2015). Perceptions and dietary intake of self-described healthy and unhealthy eaters with severe mental illness. Community Ment. Health J..

[71] Ekelund U., Steene-Johannessen J., Brown W.J., Fagerland M.W., Owen N., Powell K.E., Bauman A., Lee I.M. (2016). Does physical activity attenuate, or even eliminate, the detrimental association of sitting time with mortality? A harmonised meta-analysis of data from more than 1 million men and women. Lancet.

[72] Ciccone J., Woodruff S.J., Fryer K., Campbell T., Cole M. (2013). Associations among evening snacking, screen time, weight status, and overall diet quality in young adolescents. Appl. Physiol. Nutr. Metab..

[73] Niermann C.Y.N., Spengler S., Gubbels J.S. (2018). Physical Activity, Screen Time, and Dietary Intake in Families: A Cluster-Analysis with Mother-Father-Child Triads. Front. Pub. Health.

[74] Hall K.D., Heymsfield S.B., Kemnitz J.W., Klein S., Schoeller D.A., Speakman J.R. (2012). Energy balance and its components: Implications for body weight regulation. Am. J. Clin. Nutr..

[75] Hill J.O., Wyatt H.R., Melanson E.L. (2000). Genetic and environmental contributions to obesity. Med Clin. North Am..

[76] Armon G., Shirom A., Berliner S., Shapira I., Melamed S. (2008). A prospective study of the association between obesity and burnout among apparently healthy men and women. J. Occup. Heal. Psychol..

[77] Piercy K.L., Troiano R.P., Ballard R.M., Carlson S.A., Fulton J.E., Galuska D.A., George S.M., Olson R.D. (2018). The Physical Activity Guidelines for AmericansPhysical Activity Guidelines for AmericansPhysical Activity Guidelines for Americans. JAMA.

[78] Cook C.E., George S.Z., Keefe F. (2018). Different interventions, same outcomes? Here are four good reasons. Br. J. Sports Med..

[79] Grimby G., Borjesson M., Jonsdottir I.H., Schnohr P., Thelle D.S., Saltin B. (2015). The “Saltin-Grimby Physical Activity Level Scale” and its application to health research. Scand. J. Med. Sci. Sports.

[80] Tolkien K., Bradburn S., Murgatroyd C. (2018). An anti-inflammatory diet as a potential intervention for depressive disorders: A systematic review and meta-analysis. Clin. Nutr..

[81] Hagstromer M., Oja P., Sjostrom M. (2006). The International Physical Activity Questionnaire (IPAQ): A study of concurrent and construct validity. Public Health Nutr..

[82] Maslach C., Jackson S., Leiter M. (1997). The Maslach Burnout Inventory Manual.

[83] Chastin S.F.M., Schwarz U., Skelton D.A. (2013). Development of a Consensus Taxonomy of Sedentary Behaviors (SIT): Report of Delphi Round 1. PLoS ONE.

[84] Kim H.K., Kim M.J., Park C.G., Kim H.O. (2010). Gender differences in physical activity and its determinants in rural adults in Korea. J. Clin. Nurs..

[85] Azevedo M.R., Araújo C.L.P., Reichert F.F., Siqueira F.V., da Silva M.C., Hallal P.C. (2007). Gender differences in leisure-time physical activity. Int. J. Pub. health.

[86] Jones F., Harris P., Waller H., Coggins A. (2005). Adherence to an exercise prescription scheme: The role of expectations, self-efficacy, stage of change and psychological well-being. Br. J. Health Psychol..

[87] Beer-Borst S., Hercberg S., Morabia A., Bernstein M.S., Galan P., Galasso R., Giampaoli S., McCrum E., Panico S., Preziosi P. (2000). Dietary patterns in six european populations: Results from EURALIM, a collaborative European data harmonization and information campaign. Eur. J. Clin. Nutr..

[88] Wardle J., Haase A.M., Steptoe A., Nillapun M., Jonwutiwes K., Bellisle F. (2004). Gender differences in food choice: The contribution of health beliefs and dieting. Ann. Behav. Med..

[89] Li R., Serdula M., Bland S., Mokdad A., Bowman B., Nelson D. (2000). Trends in fruit and vegetable consumption among adults in 16 US states: Behavioral Risk Factor Surveillance System, 1990-1996. Am. J. Public Health.

[90] Purvanova R., Muros J. (2010). Gender differences in burnout: A meta-analysis. J. Vocat. Behav..

